# Systematic druggable genome-wide mendelian randomization identifies therapeutic targets for childhood asthma

**DOI:** 10.1097/MD.0000000000046718

**Published:** 2025-12-12

**Authors:** Junjie Bi, Xue Liu, Jingjing Zhang, Liqun Zhao

**Affiliations:** a Department of Gerontology, Affiliated Hospital of Shandong University of Traditional Chinese Medicine, Jinan, China; b Department of Respiratory Medicine, Affiliated Hospital of Shandong University of Traditional Chinese Medicine, Jinan, China; c Department of HR, Qilu Aerospace Information Research Institute, Jinan, China.

**Keywords:** childhood asthma, druggable target genes, Mendelian randomization

## Abstract

The management and prevention of childhood asthma remain significant challenges. Mendelian randomization (MR) has emerged as a valuable tool for identifying novel therapeutic targets. In response, we conducted a comprehensive, druggable genome-wide MR analysis to elucidate potential therapeutic targets for childhood asthma. Our study integrated genomic data concerning druggable targets, expression quantitative trait loci (eQTLs), and results from genome-wide association studies on childhood asthma. Employing the MR technique, we explored the putative causal links between genes that are targets for drugs and the development of childhood asthma. To validate these causal associations, we utilized reverse MR analyses along with colocalization techniques. Moreover, we performed enrichment analysis, mapped protein interaction networks, and executed drug prediction algorithms and molecular docking studies. These methodologies were applied to gain critical insights that could guide the development of more potent and precise therapeutic agents. We identified 35 druggable genes significantly associated with childhood asthma (including BLVRA, SLC9A3, LYZ, SRPK1, HOXA5, LYVE1, S100A9, ADORA1, RPL13, IL7R, SLFN11, SHMT1, CLN8, TOP1MT, LPAR5, RNASET2, ANK1, H6PD, DSP, CDC25B, VWF, ITK, CACNG6, ITGB7, S100A8, ADAM12, ST14, BMP6, HK2, SYK, ABCA1, ULK4, KBTBD2, SLCO4C1), with BLVRA showing promise as a target. Our research suggests that BLVRA may be a promising target for childhood asthma treatment, aiding in the prioritization of drug development for childhood asthma.

## 1. Introduction

Asthma is the most prevalent chronic respiratory disease among children, affecting approximately 10% of the global pediatric population.^[1]^Pediatric asthma is characterized by chronic airway inflammation, hyperresponsiveness, and airway remodeling. The prevalence of this disease is higher in children than in adults and continues to rise globally.^[2]^With ongoing research, it is currently understood that asthma is a complex, heterogeneous disease resulting from the interaction of numerous genetic and environmental factors.^[3]^In terms of treatment, biologics targeting downstream type 2 inflammatory pathways offer precision therapy for asthma, significantly cutting exacerbation rates, oral corticosteroid dependence and airway remodeling. FDA approved options omalizumab, IL 5 pathway inhibitors, IL 4Rα blockers and TSLP antagonists cover varied T2 sub-phenotypes and meet diverse patient needs.^[4]^ The ultra long-acting agent Depemokimab, once approved, will further streamline treatment.^[5]^ The use of inhaled corticosteroids or long-acting beta-agonists can reduce the frequency of asthma exacerbations, but they do not offer a cure.^[6,7]^Hence, there is a compelling need to persistently investigate potential therapeutic targets for the treatment of childhood asthma.

Large-scale human genetic studies offer valuable opportunities for the development of novel therapeutics for a myriad of complex diseases. This is because drug targets that are underpinned by robust genetic evidence tend to exhibit a higher probability of success throughout the drug development pipeline.^[8,9]^ In summary, druggable genes that encode proteins or regulate gene expression offer a valuable starting point for target discovery. For example, 1 study has shown that the risk of asthma is causally influenced by the levels of IL1R1, ECM1 and PDLIM4, suggesting that these 3 proteins could serve as potential drug targets for the disease.^[10]^ To date, a number of genetic findings have been translated into clinical research or practice; according to Thomson Reuters Cortellis™, compounds directed at putative asthma-risk genes are currently or have been tested in asthma clinical trials.^[11]^ At present, numerous large-scale genome-wide association studies have successfully identified a multitude of single nucleotide polymorphisms (SNPs) correlated with the risk of childhood asthma. However, the majority of these identified SNPs are located in noncoding regions or intergenic intervals, which complicates the extraction of clear and direct insights into pathogenic genes and potential drug targets from GWAS data.

Mendelian randomization (MR) is an epidemiological technique used to infer causality between a modifiable risk factor or exposure and a clinically pertinent outcome. This method uses genetic variants as instrumental variables (IVs), offering a rigorous framework for discerning causal pathways in the context of observational research.^[12,13]^MR analysis has been extensively applied for the repurposing of licensed medications^[14]^ and the identification of novel therapeutic targets^[15,16]^ by integrating summary statistics from disease-associated genome-wide association studies and expression quantitative trait locus (eQTLs) studies.Gene expression levels can be regarded as a form of lifelong exposure, and eQTLs located within the genomic regions of druggable genes are often used as proxies for such analyses.^[17,18]^

Therefore, we conducted a comprehensive, druggable genome-wide MR analysis to identify potential therapeutic targets for childhood asthma.

## 2. Methods

**Figure 1.**
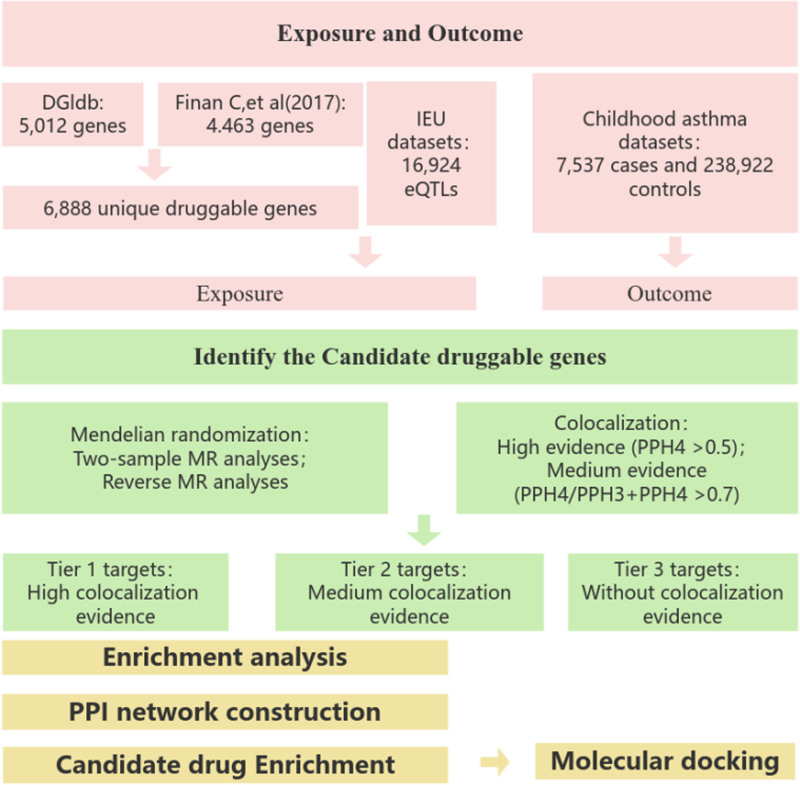
Study Overview: This investigation was structured in a sequential manner. Initially, we identified druggable genes that are potentially modifiable by pharmacological intervention. Subsequently, a 2-sample MR analysis was executed to ascertain the causal effects of druggable eQTLs on childhood asthma. To substantiate the robustness of the instrumental variable estimates, we proceeded with reverse MR analyses and colocalization analyses. In the third phase, we engaged in enrichment analysis, constructed PPI networks, and undertook drug prediction and molecular docking studies. These endeavors were aimed at furnishing insightful guidance for the development of more efficacious and precision-targeted therapeutics. DGIdb = drug–gene interaction database, eQTL = expression quantitative trait loci, MR = Mendelian randomization, PPI = protein–protein interaction.

Initially, we obtained data druggable genes and identified their eQTLs. These genes were then analyzed using a 2-sample MR approach in conjunction with GWAS data from genes that a strong association with childhood asthma. To further validate the robustness of our findings, we employed reverse MR analysis and colocalization analysis. Additionally, we performed enrichment analysis, built protein networks, predicted drug targets, and conducted molecular docking for all significant genes. These comprehensive analyses aim to provide actionable insights for the development of more efficacious and targeted therapeutic interventions for childhood asthma.An overview of this study is presented in Figure 1.

### 2.1. Druggable genes

Druggable genes were identified utilizing data from the drug–gene interaction database, which is accessible at DGIdb(DGIdb,https://www.dgidb.org/),^[19]^ in conjunction with a comprehensive review.^[20]^ The DGIdb is a valuable resource that provides comprehensive insights into drug-gene interactions and assesses the feasibility of druggability.We downloaded the most recent “Interactions Data” from the DGIdb database, which was updated through November 2024. Furthermore, we incorporated a list of druggable genes reported in a review by Finan et al By integrating druggable genes from these 2 distinct sources, we expanded our dataset to encompass a more comprehensive array of druggable genes, which has been previously leveraged in previous studies.^[21,22]^ This consolidation enhances the robustness of our analysis by expanding the dataset, allowing for a more thorough exploration of potential therapeutic targets.

### 2.2. eQTL datasets

The eQTL dataset utilized in this study was sourced from the IEU open GWAS project, accessible at https://gwas.mrcieu.ac.uk/, this dataset encompasses 16,924 eQTLs.^[23]^ The dataset employed in this study is composed exclusively of European-ancestry individuals and does not include the authors’ own Asian ethnic population.

### 2.3. Childhood asthma datasets

The summary statistics data for childhood asthma patients were obtained from the FinnGen R11 study (https://www.finngen.fi/en/). The summary data included 246,459 individuals(aged < 16 years) of Finland ancestry, with 7537 cases and 238,922 controls.^[24]^

### 2.4. Mendelian randomization analysis

MR analyses were conducted using the “TwoSampleMR” package^[25]^ We selected druggable genome eQTLs as exposure data and applied strict criteria to identify IVs for MR analysis. We included only SNPs with genome-wide significance (*P* < 5 × 10^–8^, eaf > 0.01), excluded those in high linkage disequilibrium (*r*^2^ < 0.001 within 10,000 kb), and chose strong IVs on the basis of the *F*-statistic (*F* >10). These criteria ensure the reliability and validity of our IVs in MR analysis.^[26]^

In the present investigation, we adopted the inverse variance weighted (IVW) method as our principal analytical approach for conducting MR analyses, complemented by secondary methods including MR-Egger regression, the weighted median method, and simple and weighted models.^[27]^ These methods were utilized to investigate the causal relationship between druggable gene eQTLs and childhood asthma, with statistical significance defined as *P* <.05. We assessed the odds ratios for each gene to ensure directional consistency among SNPs’ effects on exposure and outcome. Inconsistencies may suggest confounding, reverse causality, or pleiotropy. We conducted pleiotropy tests using the “mr_pleiotropy_test” function, with *P* >.05 indicating no evidence of horizontal pleiotropy for SNPs strongly associated with childhood asthma.^[28]^

### 2.5. Pleiotropy and sensitivity analyses

We evaluated horizontal pleiotropy and robustness in 4 steps:

1. MR-Egger intercept; exposures with intercept *P* < .05 were excluded.
2. Cochran *Q* and *I*²; *I*² >50% and *P*-Q <.05 indicated substantial heterogeneity.
3. Leave-one-out analysis to visualize stability after sequentially dropping each SNP.
4. Directional consistency check: IVW estimates were compared with Wald-ratio estimates for single-SNP instruments, and any exposure with contradictory SNP effects was removed.

### 2.6. Reverse MR analysis

In the initial phase of our analysis, we discerned significant SNPs within the primary dataset of pediatric asthma subjects, employing rigorous selection criteria analogous to those applied for identifying druggable gene eQTLs. Subsequently, these druggable gene eQTLs were leveraged as the dependent variable in a 2-directional MR framework to assess the possibility of inverse causality. The threshold for statistical significance was set at *P* < .05.^[29]^

### 2.7. Bayesian colocalization analysis

Bayesian Colocalization analysis was performed via the “coloc” R package to explore the potential causal links between systemic druggable genes and childhood asthma.^[30]^ This analysis is predicated on 4 primary hypotheses:

1. H0: The null hypothesis posits that the SNPs at the selected locus are not associated with either exposure (eQTL) or disease (childhood asthma).
2. H1: The SNPs are associated with eQTL but not with childhood asthma.
3. H2: The SNPs are associated with childhood asthma but not with eQTL.
4. H3: The SNPs are associated with either eQTL or childhood asthma, involving distinct SNPs for each.
5. H4: The SNPs are associated with both eQTL and childhood asthma, indicating a shared genetic basis.^[31,32]^

These hypotheses guide the interpretation of the colocalization results, providing a structured framework to assess the genetic overlap between druggable gene expression and childhood asthma susceptibility.

Significant druggable genes were stratified into 3 distinct tiers based on the strength of colocalization evidence.^[33]^ Tier 1 targets comprised genes with strong support for colocalization, defined by a posterior probability of H4 being >0.5 (PPH4 > 0.5). Tier 2 targets included genes with moderate support for colocalization, characterized by a ratio of PPH4 to the sum of PPH3 and PPH4 exceeding 0.7 (PPH4/[PPH3 + PPH4] > 0.7).^[34]^ The remaining genes were designated as Tier 3 targets.^[35]^ Subsequent analyses focused on Tier 1 and Tier 2 targets for their potential implications in druggability and association with childhood asthma.

### 2.8. Enrichment analysis

To elucidate the functional attributes and biological significance of the preselected prospective druggable genes, we employed the R package “clusterProfiler” (version 4.10.1)^[36]^ to conduct extensive GO and Kyoto Encyclopedia of Genes and Genomes (KEGG) enrichment analyses. The GO terms encompass 3 domains: biological process, MF, and cellular component (CC). Furthermore, KEGG pathway analysis provided insights into the metabolic pathways in which these genes participate.

### 2.9. Protein–protein interaction network construction

Protein–protein interaction (PPI) networks are pivotal for graphically representing the complex interactions among proteins encoded by druggable genes of interest. We constructed PPI networks utilizing the STRING database, accessible at https://string-db.org/, with a confidence score threshold established at 0.15 to delineate the minimum interaction score required for inclusion. Default settings were maintained for all other parameters to preserve the accuracy and reliability of the network assembly process.^[37]^

### 2.10. Candidate drug enrichment analysis

We downloaded the DSigDBv1.0 detailed data file from the drug signatures database (DSigDB, https://maayanlab.cloud/DSigDB),^[38]^ which contains comprehensive associations between the aforementioned genes and compounds. To ascertain which drugs within the drug signature database have a significant association with gene sets related to pediatric asthma, we conducted an enrichment analysis using the `enricher` function. This approach allowed us to identify compounds that may be significantly linked to the genetic underpinnings of childhood asthma.

### 2.11. Molecular docking

We performed molecular docking analyses to evaluate the binding energies and interaction patterns between potential drugs and their corresponding targets.This methodology enables the identification of ligands exhibiting high binding affinities and favorable interaction characteristics, thereby facilitating the prioritization of drug targets for subsequent experimental validation and optimizing the design of potential drug candidates. Structural data for the compounds were sourced from the PubChem compound database, accessible at https://pubchem.ncbi.nlm.nih.gov/,^[39]^ a reputable source for chemical structures and properties. The structural data of proteins were retrieved from the protein data bank, which is accessible at http://www.rcsb.org/.

The top ten pertinent drugs and their corresponding target proteins were selected for molecular docking analysis utilizing the CADD database (http://autodock.scripps.edu/).^[40,41]^ This process facilitated the acquisition of the final structures of 5 proteins and 6 drugs, providing valuable insights into their potential interactions.

## 3. Results

### 3.1. Druggable genome

After integrating the druggable genes from the DGIdb and previous reviews, we obtained 6888 unique druggable genes named by the Human Genome Organization Gene Nomenclature Committee for subsequent analysis. See Supplementary Material 1, Supplemental Digital Content, https://links.lww.com/MD/Q969 for details.

### 3.2. Candidate druggable genes

**Figure 2.**
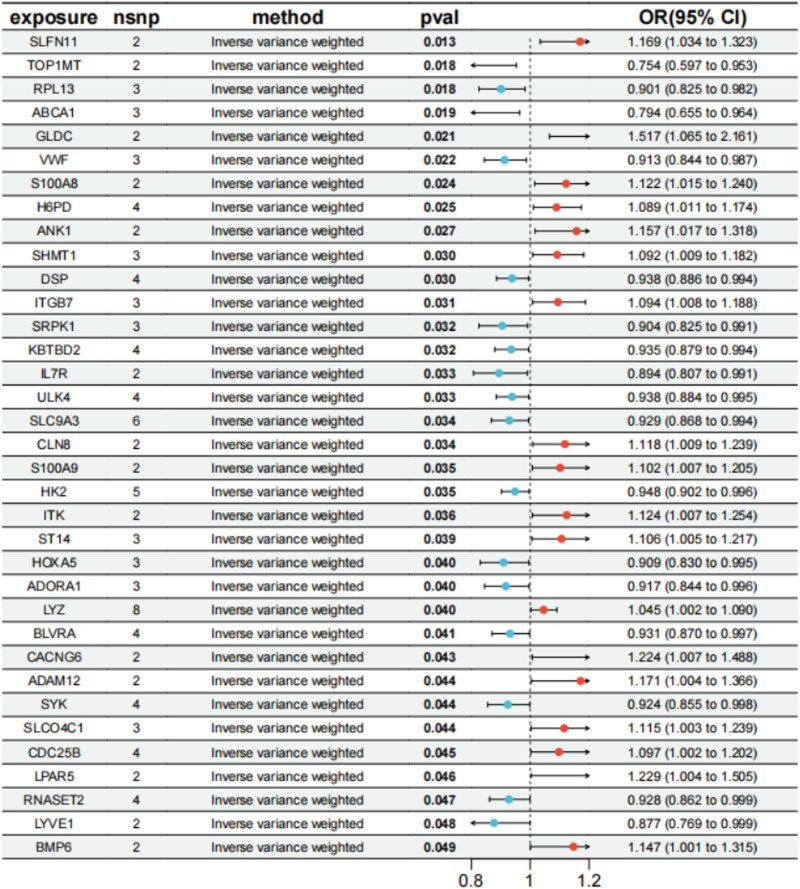
Forest plot of 35 significant genes associated with childhood asthma.

We conducted MR analysis and identified 35 genes significantly associated with childhood asthma (Fig. 2). The detailed results for the IVs and comprehensive outcomes of the MR analysis are presented in Supplementary Materials 2–3, Supplemental Digital Content, https://links.lww.com/MD/Q969.

To mitigate the potential for reverse causality between the 35 druggable gene targets and childhood asthma, we performed reverse MR analysis. In this analysis, childhood asthma was considered the exposure, whereas the 35 eQTLs of druggable genes served as the outcome. The IVW method was employed as the primary analytical approach. Our findings indicated that none of the genes exhibited a bidirectional causal relationship.

### 3.3. Bayesian colocalization

**Figure 3.**
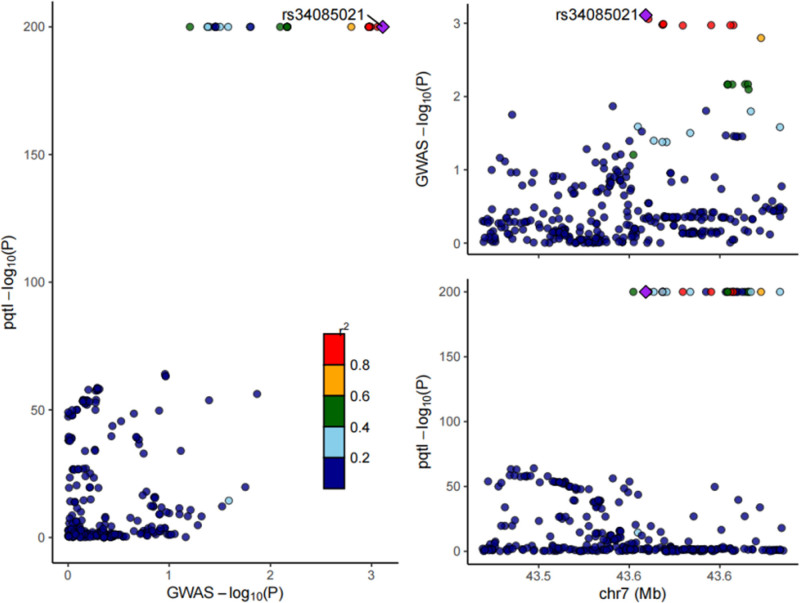
Regional locus zoom plot of the associations of SNPs with BLVRA locus. SNPs = single nucleotide polymorphisms

We conducted colocalization analyses to further assess the likelihood that the causal associations were influenced by shared genetic variants. Strong evidence of colocalization was observed between BLVRA and childhood asthma, categorizing it as a tier 1 target (PPH4 = 0.567; see Table 1 and Fig. 3).

**Table 1:** Colocalization results of 35 significant genes from blood.

Symbol	PP.H4	PP.H4/PPH3 + PPH4	Targets
BLVRA	0.57	0.98	Tier 1 targets
SLC9A3	0.03	0.70	Tier 2 targets
LYZ	0.16	0.93	Tier 2 targets
SRPK1	0.08	0.78	Tier 2 targets
HOXA5	0.02	0.73	Tier 2 targets
LYVE1	0.07	0.85	Tier 2 targets
S100A9	0.04	0.70	Tier 2 targets
ADORA1	0.29	0.85	Tier 2 targets
RPL13	0.05	0.76	Tier 2 targets
IL7R	0.05	0.72	Tier 2 targets
SLFN11	0.08	0.76	Tier 2 targets
SHMT1	0.03	0.76	Tier 2 targets
CLN8	0.06	0.78	Tier 2 targets
TOP1MT	0.07	0.81	Tier 2 targets
LPAR5	0.08	0.85	Tier 2 targets
RNASET2	0.01	0.33	Tier 3 targets
ANK1	0.03	0.43	Tier 3 targets
H6PD	0.02	0.48	Tier 3 targets
DSP	0.04	0.62	Tier 3 targets
CDC25B	0.02	0.47	Tier 3 targets
VWF	0.02	0.41	Tier 3 targets
ITK	0.04	0.49	Tier 3 targets
CACNG6	0.02	0.56	Tier 3 targets
ITGB7	0.04	0.60	Tier 3 targets
S100A8	0.04	0.68	Tier 3 targets
ADAM12	0.02	0.13	Tier 3 targets
ST14	0.05	0.55	Tier 3 targets
BMP6	0.02	0.41	Tier 3 targets
HK2	0.02	0.59	Tier 3 targets
SYK	0.04	0.48	Tier 3 targets
ABCA1	0.08	0.41	Tier 3 targets
ULK4	0.06	0.66	Tier 3 targets
KBTBD2	0.04	0.68	Tier 3 targets
SLCO4C1	0.04	0.62	Tier 3 targets

Additionally, we identified genes, including SLC9A3, LYZ, SRPK1, HOXA5, LYVE1, S100A9, ADORA1, RPL13, IL7R, SLFN11, SHMT1, CLN8, TOP1MT, and LPAR5, that demonstrated moderate evidence of colocalization (Table 1). These genes were classified as tier 2 targets. Genes with weaker colocalization evidence were designated as tier 3 targets. Overall, our MR and colocalization analyses identified 15 candidate target genes – BLVRA, SLC9A3, LYZ, SRPK1, HOXA5, LYVE1, S100A9, ADORA1, RPL13, IL7R, SLFN11, SHMT1, CLN8, TOP1MT, and LPAR5 – that indicate a shared genetic susceptibility to childhood asthma.For more details, see Supplementary Material 4, Supplemental Digital Content, https://links.lww.com/MD/Q969.

### 3.4. Enrichment analysis

**Figure 4.**
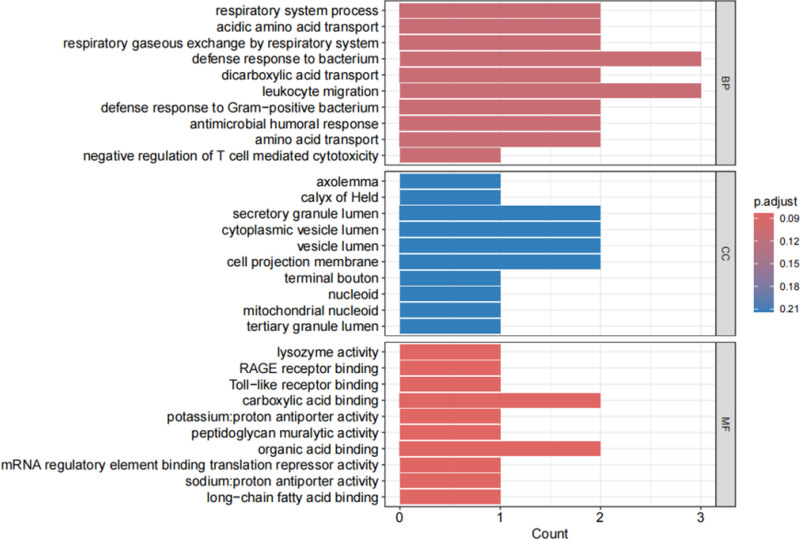
GO enrichment results for 3 terms. GO = gene ontology.

Through gene ontology (GO) analysis of the 15 potential target genes, we identified that these targets are predominantly implicated in CCs associated with defense responses to bacteria (GO:0042742) and leukocyte migration (GO:0050900). The principal molecular functions (MFs) encompassed carboxylic acid binding (GO:0031406) and organic acid binding (GO:0043177) (Fig. 4). This analysis provides insights into the biological roles of these targets and their potential relevance to the pathophysiology of childhood asthma.

**Figure 5.**
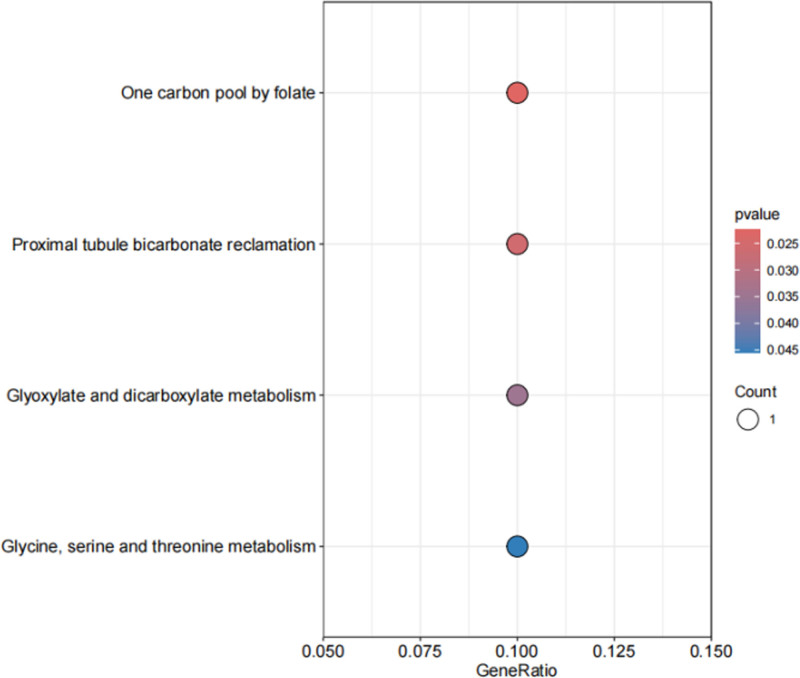
KEGG enrichment results. KEGG = Kyoto encyclopedia of genes and genomes.

In an effort to explore the therapeutic pathways potentially linked to druggable genes significant in pediatric asthma, we performed a KEGG pathway analysis. This analysis elucidated that the genes under investigation were significantly enriched in pathways, including 1 carbon pool by folate (hsa00670) and proximal tubule bicarbonate reclamation (hsa04964), as illustrated in Figure 5. These results offer critical insights into the molecular underpinnings of childhood asthma and may guide the formulation of targeted therapeutic interventions.

### 3.5. PPI network construction

**Figure 6.**
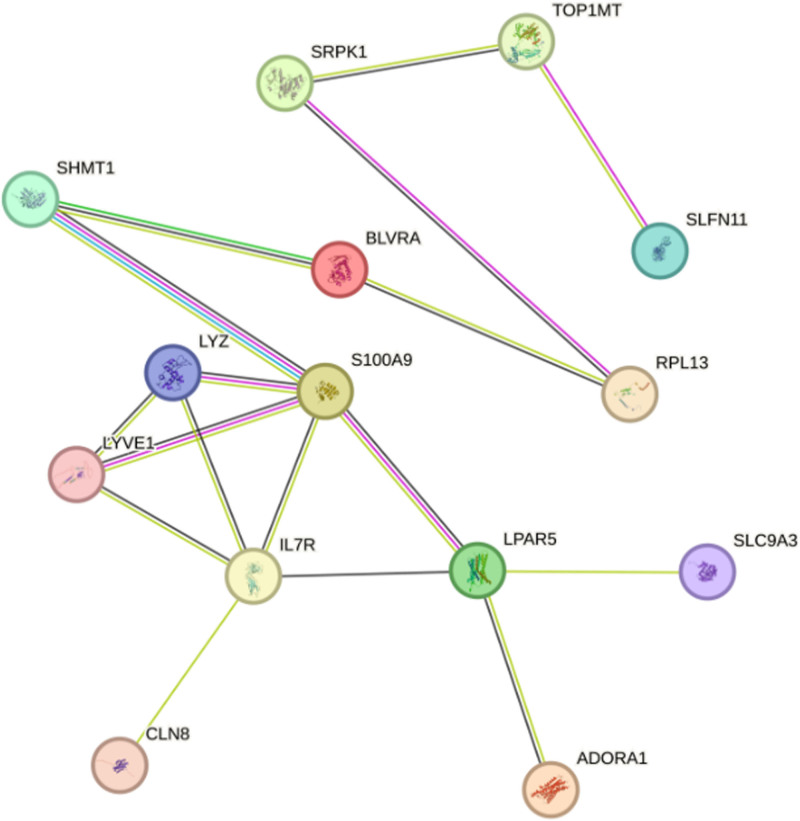
PPI network built with STRING. PPI = protein–protein interaction.

We submitted 15 drug target genes to the STRING database to construct a PPI network. The resulting network, as illustrated in Figure 6, comprised 14 nodes and 17 edges, delineating the complex protein interaction pathways.

### 3.6. Candidate drug enrichment analysis

**Figure 7.**
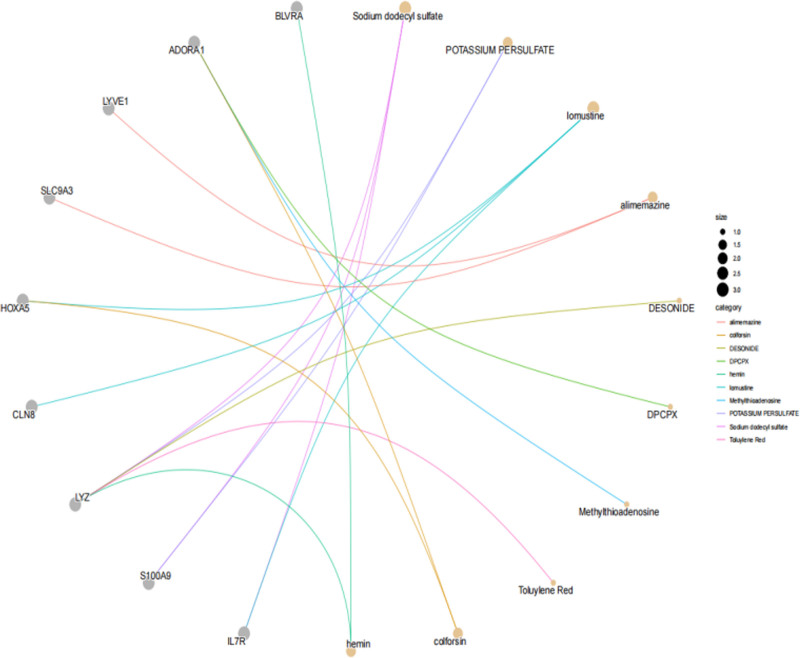
Candidate drug enrichment analysis results.

Using the DSigDB, we conducted an analysis to predict potentially effective intervention drugs for childhood asthma. The top 15 candidate drugs were shortlisted based on their associated P-values, as presented in Table 2 and Figure 7. Our findings highlighted sodium dodecyl sulfate, POTASSIUM PERSULFATE, and lomustine as the 3 most significant drugs, which are associated with the gene sets IL7R/S100A9/LYZ, S100A9/LYZ, and IL7R/CLN8/HOXA5. Additionally, hemin, which interacts with the tier 1 target BLVRA, has emerged as a notable candidate for drug intervention.

**Table 2:** Candidate drug suggested by DSigDB.

Drug name	*P*-value	*P*.adjust	genes
Sodium dodecyl sulfate	.00	.02	IL7R/S100A9/LYZ
Potassium persulfate	.00	.02	S100A9/LYZ
Lomustine	.00	.10	IL7R/CLN8/HOXA5
Alimemazine	.00	.10	SLC9A3/LYVE1
Desonide	.01	.10	LYZ
DPCPX	.01	.10	ADORA1
Methylthioadenosine	.01	.10	ADORA1
Toluylene red	.01	.10	LYZ
Colforsin	.01	.10	HOXA5/ADORA1
Hemin	.01	.10	LYZ/BLVRA
Acridin-9-amine hydrochloride	.01	.10	LYZ
Rottlerin	.01	.10	SRPK1
Sodium chloride	.01	.10	SLC9A3/LYZ
Aminophylline	.01	.10	ADORA1
Beta-dextrin	.01	.10	LYZ

### 3.7. Molecular docking

**Figure 8.**
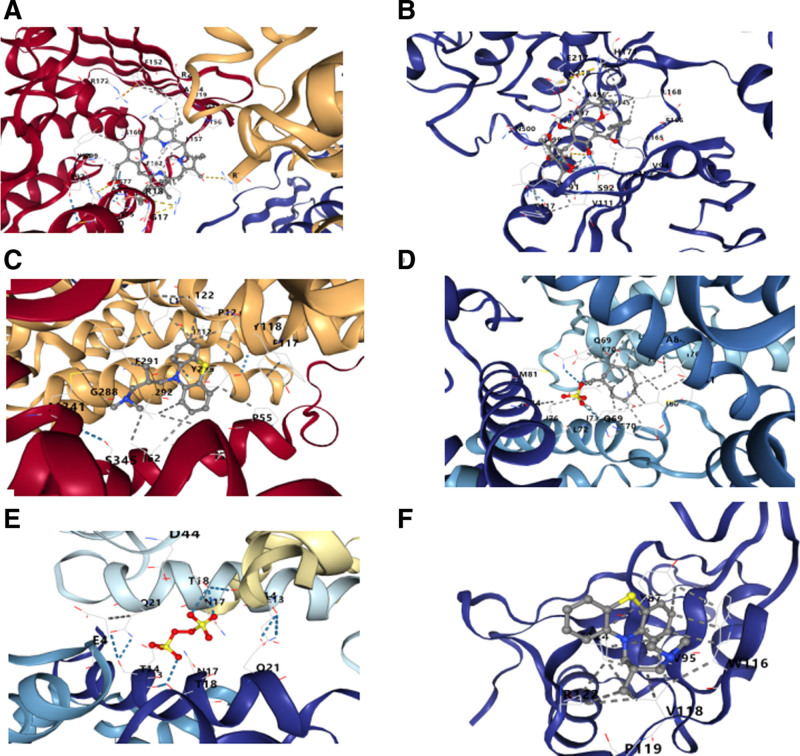
Molecular docking results of available proteins and drugs. (A) BLVRA docking hemin,(B) SRPK1 docking Rottlerin, (C) SLC9A3 docking alimemazine, (D) S100A9 docking sodium dodecyl sulfate, (E) S100A9 docking potassium persulfate, (F) LYVE1 docking alimemazine.

We performed a comprehensive analysis to delineate the binding sites and molecular interactions of the top 10 candidate drugs with their corresponding gene-encoded proteins, quantifying the binding energy for each interaction. This analysis resulted in 6 successful docking outcomes, which are presented in detail in Table 3. The specific docking amino acid residues and hydrogen bond lengths are graphically represented in Figure 8. Notably, the complexes of BLVRA with hemin and SRPK1 with Rottlerin exhibited the most favorable binding energies, reaching as low as −9.2 kcal/mol, indicative of high binding affinity and stable conformational interactions.

**Table 3:** Outcomes of molecular docking for existing protein-drug interactions.

Target	Drug	Binding energy (kcal/mol)
BLVRA	hemin	−9.2
SRPK1	Rottlerin	−9.2
SLC9A3	alimemazine	−8
S100A9	Sodium dodecyl sulfate	−6.7
S100A9	Potassium persulfate	−5.5
LYVE1	alimemazine	−5.4

## 4. Discussion

In this study, we utilized MR to integrate existing druggable gene targets with genome-wide association study data for pediatric asthma, resulting in the identification of 35 genes associated with childhood asthma. Based on the results of the collocation analysis, these genes were categorized into 1 primary target, 14 secondary targets, and 20 tertiary targets.

Furthermore, we executed enrichment analyses and generated a PPI network encompassing both primary and secondary targets. This approach was employed to elucidate the biological relevance and interaction dynamics of these drug targets. Subsequently, drug prediction models and molecular docking simulations were undertaken to substantiate the therapeutic potential of these notable druggable genes.

The biliverdin reductase A encoded by the BLVRA gene is involved in the metabolism of bilirubin. The mechanism of action involves the binding of the BLVRA protein to the heme oxygenase-1 (HO-1) protein, thereby modulating the catalytic activity of HO-1. Additionally, it induces the expression of HMOX1 through oxidative stress.^[42]^A prospective cohort study, conducted across multiple centers in the United States between 1959 and 1965, identified a correlation between neonatal hyperbilirubinemia and an elevated risk of asthma during childhood.^[43]^ Further research indicated that moderate levels of hyperbilirubinemia are associated with an increased risk of asthma, while no correlation was detected at higher levels.^[44]^ Numerous studies have shown an association between total serum bilirubin levels in children’s blood and the incidence of asthma, suggesting that bilirubin metabolism may play a role in respiratory health during early life.^[45]^

Recent research has suggested that oxidative stress may play a significant role in the pathogenesis of asthma.^[46]^A case report described a patient with persistent, difficult-to-control asthma who experienced complete resolution following an increase in serum bilirubin levels due to acute hepatitis B, suggesting a potential therapeutic role for antioxidant agents in asthma management.^[47]^An animal study demonstrated that bilirubin can exert a protective effect during the establishment of asthmatic animal models by reducing oxidative stress responses and promoting the secretion of IL-10, thereby modulating the immune system balance, and altering the secretion pattern of Th1/Th2/Th17-related cytokines.^[48]^In asthmatic patients, airway inflammation and oxidative stress result in increased production of free radicals, often outpacing the body’s antioxidant defenses. Bilirubin, produced by BLVRA, serves as an endogenous antioxidant that can protect cells from oxidative damage and reduce the generation of reactive oxygen species,^[49]^ thereby alleviating oxidative stress-induced injury to airways.Bilirubin effectively scavenges chemically generated peroxyl radicals, hydroxyl radicals, superoxide anion radicals, and lipid peroxides.^[50]^ In particular, under hypoxic conditions, such as 2% oxygen, which is close to physiologically relevant concentrations, the antioxidant activity of bilirubin is enhanced.^[51]^ Among liposomes, bilirubin has greater inhibitory effects on oxidation than does α-tocopherol, and its antioxidant activity is severalfold greater than that of other antioxidants, such as β-carotene, vitamin E, and vitamin C.^[52]^

BLVRA may be involved in the modulation of inflammatory responses. Bilirubin at physiological concentrations can control inflammatory reactions by inhibiting the activation of the NF-κB signaling pathway and inflammasomes. This findings suggest that bilirubin is capable of effectively suppressing the release of inflammatory cytokines, such as IL-1β and TNF-α, both in vitro and in vivo, thereby improving survival rates in murine models of sepsis.^[53]^Leukocyte migration from the bloodstream into tissues is a critical process for immune surveillance and the inflammatory response. In vivo studies have demonstrated that the administration of the antioxidant bilirubin effectively inhibits VCAM-1-dependent leukocyte migration into the lungs in experimental asthma models.^[54]^Studies have indicated that during periods of asthma exacerbation, there is a significant decrease in both total and indirect bilirubin levels compared with periods of asthma control.^[55]^In chronic obstructive pulmonary disease (COPD), bilirubin levels are negatively correlation with inflammatory markers, suggesting that higher bilirubin levels may serve as an independent protective factor against COPD and its acute exacerbations.^[56]^Bilirubin nanoparticles have demonstrated significant protective effects in models of sepsis-induced acute lung injury, capable of reducing pathological changes in lung tissue and mitigating the progression of inflammation.^[57]^Bilirubin has also shown antioxidant and immunomodulatory effects in inflammatory bowel disease, where it can inhibit digestive proteases and modulate the gut microbiota. This further supports its potential application in inflammation-related diseases.^[58]^Given the antioxidant and anti-inflammatory properties of bilirubin,^[46,59]^ it may serve as a potential diagnostic marker and therapeutic target in the clinical management of pediatric asthma in the future.

In this study, DSigDB predicted 15 potential drugs for childhood asthma, but current clinical research is mainly focused on aminophylline. Aminophylline is a traditional bronchodilator that has been the subject of ongoing debate regarding its use in the treatment of asthma, particularly in the context of acute asthma attacks in children.^[60,61]^Several studies have demonstrated the efficacy of aminophylline in the treatment of acute pediatric asthma exacerbations. Despite concerns regarding its safety and side effects, aminophylline may be considered a second-line therapeutic option when children exhibit an inadequate response to inhaled bronchodilators and oral corticosteroids. Evidence suggests that aminophylline can improve pulmonary function and reduce hospital stay, albeit with a potential increased risk of nausea and vomiting.^[62–64]^The 2005 Cochrane review indicated that, compared with placebo, aminophylline improved pulmonary function in children; however, there was no significant reduction in hospitalization duration or the number of bronchodilator treatments required.^[65]^Some studies have shown that the combined use of aminophylline with other medications, such as bronchodilators, can significantly enhance therapeutic efficacy.^[66]^

Hemin is associated with the tier 1 target BLVRA and is capable of inducing the expression of HO enzymes, particularly HO-1.^[67]^ HO-1 plays a crucial role in regulating the balance between T helper 1 (Th1) and T helper 2 (Th2) cells by increasing the proportion of naturally occurring regulatory T lymphocytes (CD4 + CD25High) and enhancing their functionality. This modulation helps to counteract allergic airway inflammation.^[68]^Hemin is capable of modulating the function of immune cells, including the inhibition of pathological Th17 cell generation and the promotion of regulatory T (Treg) cell activation. This action helps to balance the Th17/Treg cell ratio, thereby alleviating airway inflammation.^[69–71]^In a systematic review, researchers summarized the pharmacological effects of hemin in various inflammation-related diseases, including its anti-inflammatory and antioxidant properties. Studies indicate that hemin can mitigate oxidative damage by increasing the activity of antioxidant enzymes, such as superoxide dismutase (SOD), and by reducing the expression of oxidative stress markers, such as malondialdehyde (MDA) and myeloperoxidase (MPO).^[72]^In an acute lung injury model, hemin treatment significantly reduced the expression of pro-inflammatory factors such as NF-kB, caspase-1, IL-1β, IL-6, and TNF-α, and increased the activity of SOD, while decreasing the expression of MDA, MPO, and reactiveROS.^[73–76]^

Colforsinas, an activator of adenylyl cyclase, is capable of increasing the intracellular level of cyclic adenosine monophosphate (cAMP).^[77]^By activating the cAMP signaling pathway, colforsin can directly act on airway smooth muscle, leading to its relaxation, alleviating bronchospasm, and improving airflow. Research advancements indicate that modulating the cAMP pathway has potential application value in the prevention and treatment of respiratory diseases. Increasing intracellular cAMP levels may help alleviate symptoms of asthma, COPD, and other respiratory conditions.^[78]^Methylthioadenosine, an endogenous metabolic product, has the ability to modulate the function of immune cells. For example, it can inhibit excessive activation of macrophages and reduce the release of inflammatory mediators, thereby contributing to the alleviation of airway inflammation.^[79]^

The current study has several notable strengths that enhance its validity and scientific impact. Firstly, we employed MR and colocalization analyses, which are robust methodologies that utilize genetic variants to establish causal links between drug target genes and childhood asthma. This approach is instrumental in mitigating biases stemming from confounding factors and reverse causality, thereby fortifying causal inferences. Secondly, our research represents an inaugural application of MR design specifically aimed at druggable target genes for childhood asthma. This focus is of paramount importance given the protracted and costly drug development process. Through a data-driven methodology, we are able to pinpoint potential novel drug targets, offering a promising avenue for advancing childhood asthma treatments.

Enrichment analyses and PPI studies have been instrumental in deciphering the functional roles and regulatory interactions of the target genes, thereby mapping out potential therapeutic avenues for pediatric asthma. The therapeutic potential of these genes is further highlighted by drug prediction models, and the robust binding affinities observed in molecular docking studies confirm their suitability as drug targets.

Our investigation offers an in-depth examination of the genetic factors implicated in pediatric asthma, extending from the identification of druggable gene targets to an assessment of their therapeutic potential. This holistic methodology has significantly broadened our understanding of the genetic substrate of childhood asthma and has laid the groundwork for the development of more targeted and efficacious treatment strategies for asthma, underpinned by robust evidence.

Despite yielding novel findings, our study is subject to certain inherent limitations. Firstly, the interpretation of genetic variation data to predict the therapeutic efficacy of potential drugs for childhood asthma is hampered by the disease’s complex etiology, which involves a multitude of genes, factors, and pathogenic pathways. Consequently, MR is more adept at establishing the causal direction rather than assessing the strength of associations. Secondly, the scope of IVs in eQTL MR analyses is often constrained, with the majority of analyses considering no more than 3 SNPs, potentially impacting the robustness of the MR outcomes. Thirdly, even after conducting comprehensive assessments for heterogeneity and pleiotropy, the possibility that horizontal pleiotropy could influence our results cannot be completely ruled out. Fourthly, our study’s reliance on GWAS databases, which predominantly consist of data from individuals of European descent, may restrict the applicability of our findings to populations of different ethnic backgrounds. Lastly, the precision of molecular docking studies is significantly dependent on the quality of the protein structures and ligands that are utilized. While this technique has helped to identify potential drug targets, it does not establish their clinical effectiveness. Consequently, additional experimental confirmation and clinical studies are imperative to ascertain the therapeutic viability of the targets we have identified.

## 5. Conclusions

This study identified 1 robust druggable gene (BLVRA) and 34 candidate druggable genes for childhood asthma. The results present hopeful avenues for the advancement of more potent therapies for childhood asthma, which could also lead to a reduction in drug development expenses. By underscoring the substantial link between these targetable genes and pediatric asthma, this study adds considerable value to the scientific domain. It is justified to conduct further clinical studies on medications aimed at these genes in the forthcoming period.

## Acknowledgments

We thank our families for their unwavering support and companionship throughout this study. Special gratitude goes to our newest family member, born in 2025, whose every milestone coincided with the creation of this manuscript.

## Author contributions

**Conceptualization:** Junjie Bi, Jingjing Zhang.

**Data curation:** Junjie Bi, Jingjing Zhang.

**Formal analysis:** Junjie Bi, Liqun Zhao.

**Funding acquisition:** Xue Liu, Jingjing Zhang, Liqun Zhao.

**Investigation:** Liqun Zhao.

**Methodology:** Jingjing Zhang.

**Resources:** Xue Liu.

**Validation:** Xue Liu.

## Supplementary Material
